# A Data-Driven Framework for Team Formation for Maintenance Tasks

**DOI:** 10.36001/ijphm.2021.v12i1.2930

**Published:** 2021-03-24

**Authors:** Maya Reslan, Emily M. Hastings, Michael P. Brundage, Thurston Sexton

**Affiliations:** 1 National Institute of Standards and Technology, Gaithersburg, MD 20814, USA; 2 University of Illinois, Urbana, IL 61801, USA

## Abstract

Even as maintenance evolves with new technologies, it is still a heavily human-driven domain; multiple steps in the maintenance workflow still require human expertise and intervention. Various maintenance activities require multiple maintainers, all with different skill sets and expertise, and from various positions and levels within the organization. Responding to maintenance requests, training exercises, or executing larger maintenance projects all can require maintenance teams. Having the correct assortment of individuals both in terms of skills and management experience can help improve the efficiency of these maintenance tasks. This paper presents a workflow for creating teams of maintainers by adapting accepted practices from the human-computer interaction (HCI) community. These steps provide a low-cost solution to help account for the needs of maintainers and their management, while matching skills of the maintainers with the needs of the activity.

## 1. Introduction

Maintenance is an essential task in any manufacturing company. When maintenance is performed incorrectly, e.g., performing the wrong maintenance action, or spending too long on a maintenance task, the plant’s overall productive capacity can be decreased up to 20 %. These resulting equipment failures cost the production industry around $50 billion a year (ATOM, 2018).

The maintenance workflow involves many steps, including: 1) Selecting and Prioritizing maintenance jobs, 2) Planning the maintenance tasks, 3) Scheduling maintenance jobs, 4) Executing and 5) Completing the job, and 6) Analyzing the work (Brundage et al., 2019). Many of these steps require teams of different maintainers and managers from all levels of the organization. For example, when planning a large maintenance job, a technician, an engineer, and a maintenance planner may all work together to perform the planning steps.

This paper investigates team formation strategies from other domains to be adapted to improve maintenance operations. Research has shown that effective team formation techniques have shown that improved morale leads to more effective employees, with no negative effect on important metrics (which vary depending on the domain) (Duhigg, 2016; Edmondson, 1999).

The structure of maintenance teams is analogous to team structure in other domains where you have groups of people with different skills and backgrounds working together for a common project. Therefore, techniques for team formation and team building in these other disciplines may prove useful in the maintenance context as well. For example, fostering successful teams is an important component in higher education, where instructors must prepare students for jobs in industries that highly value the ability to work collaboratively (Robles, 2012). Standards created by agencies like the Accreditation Board for Engineering and Technology (ABET) also require students to be able to “function effectively as a member or leader of a team” (ABET, 2019) before universities can be certified.

Instructors in academia are similar to managers in the maintenance context, in that they are trying to lead students or non-managers in the successful completion of a project. However, a significant difference between academia and maintenance is the expertise and knowledge of maintainers versus students. Maintainers frequently have many years of experience in a particular domain or speciality (e.g., a plumber or mechanic), and also often have more autonomy within the company than students do in their roles. However, both maintainers and students frequently have an eagerness to learn. Maintenance is a life-long skill where maintainers constantly learn new techniques and maintain new equipment.

The framework proposed in this paper allows for newly learned skills to be explicitly incorporated in the formation of the team. The maintenance domain also has the potential to reuse teams in multiple projects over time and the ability of management to be included in teams, both of which are not common features in an academic setting. However, despite these differences, the commonalities between these two domains suggest that techniques from one field could be used to improve outcomes in the other. Therefore, this paper adapts well established team formation research shown to be successful in academia, to improve maintenance teams’ efficiency, quality, and morale.

## 2. Literature Review

Accomplishing effective team optimization involves many factors, but one of the first and most important questions that must be addressed is how teams should actually be formed. This process is a subject of research in a number of fields, including project management (Raiden, Dainty, & Neale, 2004; Tseng, Huang, Chu, & Gung, 2004), computer-supported cooperative work (CSCW) and human-computer interaction (HCI) (Gómez-Zará, DeChurch, & Contractor, 2020; Harris, Gómez-Zará, DeChurch, & Contractor, 2019), and the learning sciences (Chapman, Meuter, Toy, & Wright, 2006). Strategies include self-selection, random assignment, and criteria-based approaches, which consider information about potential team members such as demographics, skills, and learning styles when forming teams. The first two of these approaches (self-selection and random assignment), while easy to implement, tend to produce teams that are overly homogeneous and lack necessary skills for completing their assigned tasks (Bacon, Stewart, & Silver, 1999; Chapman et al., 2006; Jalajas & Sutton, 1984; Janis, 1982). Criteria-based approaches address this problem by, for example, allowing instructors to distribute students with necessary skills across teams. Unlike self-selection, criteria-based approaches also ensure that every potential member is able to join a team, regardless of factors like how many people they know in their organization (Jahanbakhsh, Fu, Karahalios, Marinov, & Bailey, 2017).

In academia, instructors are increasingly turning to algorithmic tools such as the Comprehensive Assessment for Team-Member Effectiveness (CATME) Team-Maker (Layton, Loughry, Ohland, & Ricco, 2010) to help implement criteria-based approaches to team formation, especially as course enrollments grow. Tools like CATME Team-Maker are also applicable in other contexts that utilize teams, including massive open online courses (MOOCs), online labor markets like Amazon’s Mechanical Turk (MTurk), and in the maintenance domain. In general, algorithmic tools can help to streamline the team formation process for both instructors and students, although they tend to leave little room for student input into the team formation process (Hastings et al., 2020; Jahanbakhsh et al., 2017), and often rely on input data of uncertain accuracy self-reported by students (Alamri & Bailey, 2018).

A current line of research in the HCI and CSCW communities and other areas examines the use of these algorithmic approaches to team formation in more detail, primarily in project-based undergraduate courses. One such work is LIFT: the Learner Involvement in Forming Teams framework (Hastings et al., 2020), which we adapt for a maintenance context in this paper. In particular, this strand of research examines stakeholder perceptions of the strengths and weaknesses of existing tools, and aims to create a more learner-centered approach to algorithmic team formation, in which students have more control over a process from which they have heretofore been excluded. These types of cultural changes (i.e., having students or non-managers more involved in the team formation process) can not only improve morale, but can also lead to improved team performance as seen in industry (Raiden et al., 2004).

**Figure 1.**
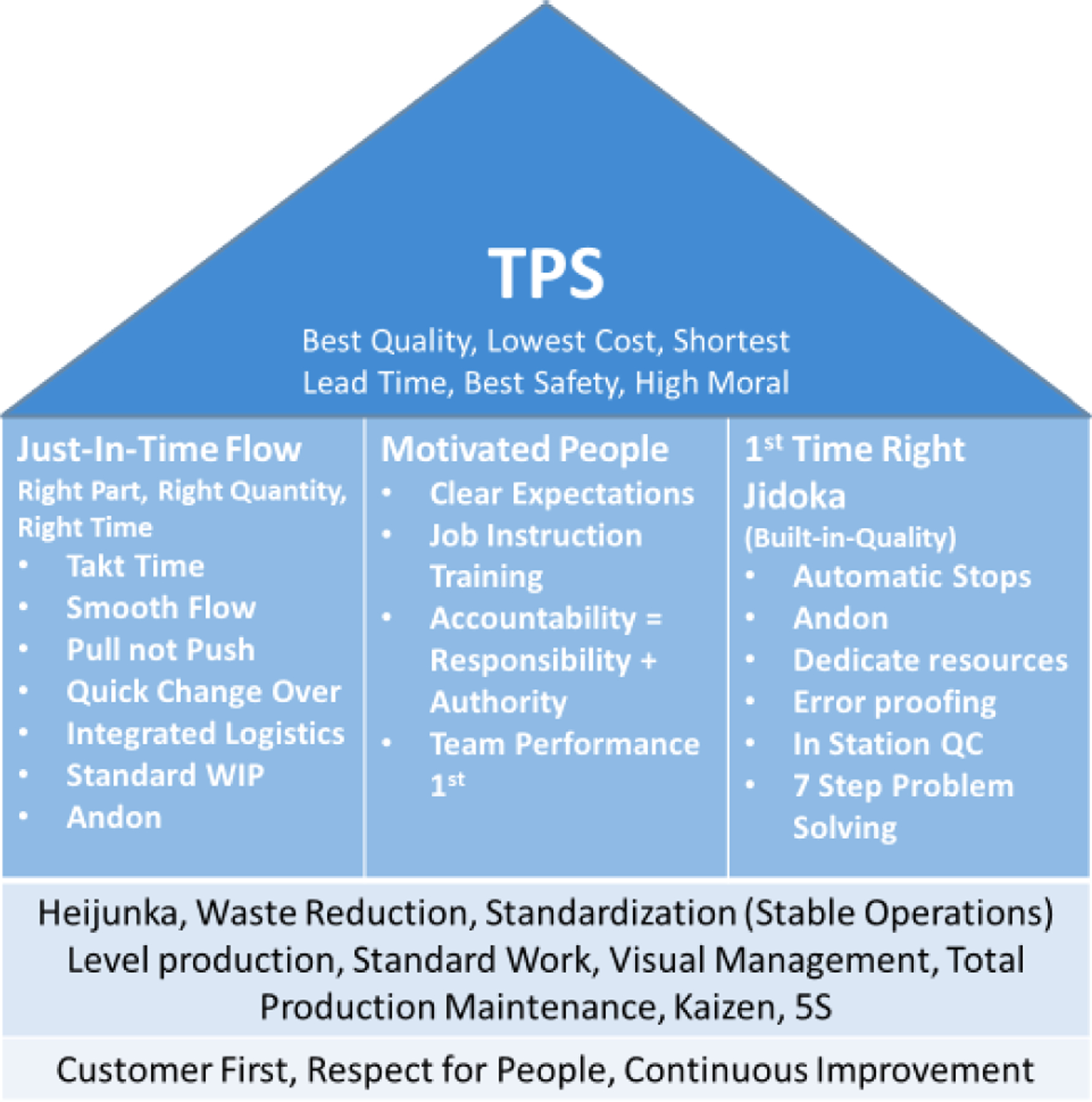
House of Toyota Production System adapted from (Liker & Morgan, 2006).

In the industrial setting, culture can have important ramifications. The term ‘organizational culture’ is defined as the standards, behaviours, and beliefs that contribute to the exceptional social and psychological atmosphere of an organization and can be expressed through the organization’s tasks and responsibilities, how they are being done, and by whom (Baqlah, 2017). In a world where competing companies have access to the same technology and innovations, improving organizational culture can improve performance compared to competitors. When the concept of organizational culture was first introduced, companies like IBM, General Motors, and IE DuPont Nemours were recognized for their strong sense of identity and success that constantly communicated their culture to their employees, which led to a large competitive advantage and success (Waterman & Peters, 1982). On the other hand, it was evident that many companies where their leaders did not acknowledge the power of culture ultimately lost some level of competitive advantage. Company culture not only shapes employees’ attitudes and behavior, but also company performance. Organizational culture is imperceptible yet significant and a powerful force that shifts the entire organization either negatively or positively (Taherimashhadi & Ribas, 2018). The road to cultural improvement includes incremental change, continuous improvement, and understanding the dynamism of the company along with the employees and their needs, which leads to meeting the organization’s objectives (Uddin, Luva, & Hossian, 2013). These types of improvements have been prevalent in manufacturing since the introduction of lean manufacturing, which started with the Toyota Production System (TPS). The vital principles of lean manufacturing, are shown in Fig. 1 as the House of TPS.

The ultimate goals of TPS are priority to the customer, respect for the employees, and continuous improvement. The different areas of the “house” in the figure show the methods used to achieve each goal. TPS’s main concept is reduction of cost production through elimination of waste, which means that anything other than the minimum amount of equipment, material, parts and working time vital to production is just a surplus that only raises costs. As part of reducing waste, lean encourages the full use of a workers’ capabilities, where workers are treated with respect, which in turn encourages workers to be trusted with greater responsibility and automony in order to display their capabilities (Wagner, Herrmann, & Thiede, 2017). As an example of this increased responsibility, Toyota aimed to build a company that has “visible control”, where the detection of problems is not limited to only the managers, but is the responsibility of all employees. The workers responded and took positive steps to improve and eliminate problems without any push from their managers; and increased their contributions to the company (Sugimori, Kusunoki, Cho, & UCHIKAWA, 1977). When ideas are brought up to pull a company out of the past and onto the road of improvement, performance and efficiency are increased. Any person in a company can have a major impact on its performance, regardless of whether that person is the CEO or an intern. This cultural focus on including perspectives from multiple levels of the organization is key to effective team formation.

A focus on organizational culture at all organizational levels should be a prerequisite for the implementation of continuous improvement (Bhasin & Burcher, 2006; Schein, 2010). Therefore, focusing on organizational culture is more vital than ever before (Schein, 2010). This paper focuses on improving organizational culture through the formation of better teams by accounting for perspectives at different levels of the organization.

The rest of the paper is structured as follows. Section 3 discusses the LIFT methodology (Hastings et al., 2020), which is the basis for the team formation procedure proposed here for maintenance. Section 4 presents the steps for maintenance team formation and discusses hypothetical use cases for each step. Lastly, Section 5 provides conclusions and next steps for this work.

## 3. LIFT Methodology

The Learner Involvement in Forming Teams (LIFT) workflow (Hastings et al., 2020) provides a method to integrate stakeholder voices into algorithmic team formation. LIFT was originally designed to account for student and professor perspectives in team formation for group projects in courses. In LIFT, the configuration of the criteria and weights used in the team formation tool is delegated to the students in the course, instead of being determined solely by the instructor. This approach allows the students to have more input into a process affecting their team experience, learning, and grades, and is grounded in theories of crowdsourcing and collective intelligence, including prior applications of crowdsourcing techniques in learning environments e.g., (Basharat, 2016; Glassman, Lin, Cai, & Miller, 2016; Glassman & Miller, 2016; Kim, 2015; Lasecki et al., 2012; Li & Mitros, 2015; Pavel, Goldman, Hartmann, & Agrawala, 2016; Weir, Kim, Gajos, & Miller, 2015; Williams et al., 2016)).

As originally proposed, the LIFT workflow consists of three main steps:

### Discuss

Students discuss the criteria that they think are important for forming teams in the course. In the implementation studied in (Hastings et al., 2020), this discussion occurred on an online forum seeded with a list of the criteria that already existed in the chosen team formation tool. Students were asked to make at least 3 contributions to the discussion, where a contribution was either a) proposing a criterion (novel or existing) and explaining why they think it should be included or b) commenting on another posted criterion and discussing its strengths/weaknesses or proposing modifications.

### Vote

Students vote to determine which of the proposed criteria from the discussion phase will actually be used to form teams. The research team in (Hastings et al., 2020) created a ballot by extracting all of the criteria from the discussion and discarding duplicates, infeasible criteria, etc. Students then voted on the remaining criteria using a 5-pt Likert item (“This criterion should be included [in the tool],” −2= Strongly disagree, 2=Strongly agree). Scores were totaled for each criterion to create a ranking of the level of support, and the most popular criteria were selected for use in the tool (according to a specified threshold).

### Configure Weights

Students configure the weights for each selected criterion and the tool forms the teams. In (Hastings et al., 2020), which used the tool CATME Team-Maker (Layton et al., 2010) to form teams, students provided their personal response for each selected criterion in the tool, and at the same time provided their desired weight (magnitude from 0 to 5) and preference for whether similar/dissimilar students should be grouped (the sign of the weight) for each criterion. For example, assigning a weight of 5 to the “Schedule” criterion strongly prefers groups where students report similar schedules, whereas assigning “Shop skills” a weight of −2 would moderately prefer groups of students with varying “Shop skill” levels. The final weight used in the tool for each criterion was the floor of the mean of the magnitudes students provided, with the sign that had received the most votes. For example, if students had voted −1, 1, 2, 3, the weight used by the tool was ⎣1.75⎦ = 1.

In addition to the full workflow described in (Hastings et al., 2020), the LIFT authors also suggested several potential modifications to the process to facilitate adoption in different contexts. For example, instructors could perform the full workflow only once every few years as they become more familiar with the criteria students tend to select. A reduced workflow could be implemented in the intervening terms, for example, one where students vote on the weights used but not the criteria themselves. Additionally, teams could be formed using a set of criteria combining both student and instructor selections, incorporating the knowledge and goals of both parties.

The LIFT methodology along with these modifications are the basis for the proposed maintenance team formation workflow.

## 4. Methodology

We propose a low-cost framework for team formation for maintenance tasks adapted from LIFT. Our framework extends LIFT for use in this new context by adding a focus on continuous improvement and offering flexibility to suit the needs of maintenance stakeholders in individual companies. As discussed earlier, this team formation methodology can be useful for a variety of maintenance activities, such as schedule planning, responding to maintenance requests, working on large maintenance engineering projects, or creating teams for training exercises. Each of these activities requires a diverse team of different domain skills and expertise levels. For example, if a company needs to rebuild an entire asset during a large shutdown of a line, what is the best team? It may require having an engineer, a mechanic, an electrician, and an outside consultant. What skill levels are necessary for each? The team may also require management experience to lead the team and to provide input into the executive perspective. Do personalities matter to having a successful team? Should non-managers get a say in forming the team? Adapting the LIFT framework for maintenance helps provide solutions for these questions providing improvement to maintenance operations while also improving employee morale. We propose the following steps for effective team formation:

1. Discuss necessary criteria
2. Vote on the criteria set
3. Assess level for each criterion
4. Create teams using above steps
5. Solicit feedback on effectiveness of the teams

These steps are presented in more detail in the following subsections.

It is also important to keep in mind that it is up to the stakeholders in each individual company to decide what it means to be a good team by acknowledging what is vital and a priority for their goals. The people responsible for this decision are usually the managers, analysts, or HR depending on the company’s structure. However, this process may be unnecessary for small companies with few employees.

### 4.1. Discuss Necessary Criteria

This step involves maintainers and their managers discussing necessary criteria for a successful project. We provide some potential examples in Table 1, adapted from the criteria from the original LIFT framework in (Hastings et al., 2020). Some of these criteria could include personality traits or leadership skills, which are analogous to what would be seen in the student LIFT procedure. For example, when performing a large maintenance rebuild with multiple tradespeople, the team may require someone apt in project management. Other more specific engineering domain criteria may also be necessary, such as skills in maintenance (e.g., having someone with pipe fitting or electrical skills). Another important criterion to consider in maintenance is budget constraints, including differing cost of employees.

Most of the time, employers only take into account the technical skills when planning a team for a maintenance job; however, it is also vital to take into consideration the interpersonal skills and innate propensities of each person when forming a team to reach maximum effectiveness. Team performance highly relies on each team member’s behaviors and interpersonal interactions with one another as well as their technical expertise (Fitzpatrick & Askin, 2005). For example, many studies have been performed on personality traits and human behavior. It has been shown that personality traits influence our behavior in many situations and therefore affect how we interact with those around us and consequently how people see us. Wechsler’s studies show that personal skills and intellectual ability cannot be considered independently from drive, temperament, or emotion, which are referred to as personality traits (Wechsler, 1950). Teams where the members have diverse thinking styles and personalities achieve better outcomes than homogeneous teams (Hammer & Huszczo, 1996). Hence, a proper assessment of personality traits can be an important part of team formation.

However, using these qualitative criterion within a quantitative framework like LIFT is quite difficult. To integrate them, we need to quantitatively *measure* them in a useful way. This is a deep topic and largely outside the scope of this paper. As examples, we discuss two metrics that can be used as proxy for personality assessment, in order to include them as LIFT criterion.

**Human Brain Dominance Index (HBDI)** an assessment for the classification and description of thinking preferences of people in order to adapt those thinking preferences to communicate effectively, and improve problem solving and decision making. (Fitzpatrick & Askin, 2005)

**Kolbe Conative Index (KCI)** a psychometric system that weighs conation that is applied to shape successful teams. *Conation* is the part of the mind that manages conscious intention and aims to carry out volitional acts; it is about how people approach problem solving, organize their ideas or objects, and consume their time and energy. Multiple studies proved that this index is efficient when it comes to team performance because the team’s success is heavily connected to the balance of these conative energies inside it. (Bailey, 2002).

Metrics and techniques like these can be used if personality traits are deemed to be an important aspect of forming the maintenance team, as they can complement the more traditional “hard” skills as described above. As an example, the maintainers may want to have a mixture of hands on skills with diverse thinking styles to tackle a longer term maintenance project. The next step in this process allows maintainers and managers to vote for the criteria they find necessary for successful team formation in the next step.

### 4.2. Vote on Criteria Set

After the list of all possible criteria is discussed and debated, maintainers and managers vote on what is the subset of criteria that will be used for creating potential teams, and the relative importance of these criteria (i.e., their weights in the algorithm). For example, a manager may deem plumbing skills to be invaluable, but programming skills to be completely unnecessary. However, a maintainer may want team members to connect on a more personal level if this project is a long term endeavor. An example of potential weights for criteria of maintainers and managers is shown in Table 2.

The voting process should be transparently described, understood, and agreed upon ahead of time. Preferably, a wide range of stakeholders at varying levels of responsibility will vote, to accurately capture relevant organizational preferences as a whole. Due to hierarchical structures within the organization, some may wish to give preference to voters in positions of greater responsibility (and, therefore, accountability). However, implementers must take great care not to undermine the original intent of this procedure: namely, *team*-building. Any asymmetries in vote weighting risk eating away at perceived trust in the process. The need for cultural buy-in to the voting outcomes necessitates transparency.

In the original LIFT workflow, voting was accomplished by ranking the level of support for each proposed criterion using a 5-pt Likert scale. Criteria were scored by each individual participant, then ranked according to their aggregate score across participants. While this technique is straight-forward to implement, interpreting the strength of preferences through aggregated Likert scores presents some potential issues, such as the need to sum over ordinal scores, the need for wide agreement on precise definitions for each scale value (e.g., what does “mostly agree” really mean?) and meaningful ways to compare values (e.g., are “disapprove” and “approve” equidistant from “neutral”?). There is also a risk of individuals with strong or extraordinary preferences having an inordinate effect on results (e.g., someone that only gives low scores except in one category).

A number of other possibilities for aggregating group preferences exist that do not rely on wide agreement on ordinal scale interpretability. The choice largely depends on whether it is preferable for individuals to report their preferences as *scores*, as in the Likert scale above, or as *ranked preferences*. For scores, one such technique is *majority judgement*, which uses median scores with a tie-breaking procedure to output a robust ranking of criteria (Balinski & Laraki, 2011). The alternate use of requesting ranked user preferences instead of scores, on the other hand, enables the use of, for example, the *Kemeny-Young method* to find a complete ranking (Young & Levenglick, 1978).

### 4.3. Assess Level for Each Criterion

Once the subset of criteria and their weights are voted on and finalized, each potential team member will report their level with respect to each of the criteria, e.g., their personality type or skill at a particular task. In the original version of LIFT, students self-assessed their levels. Since student projects are often one-off endeavors, less quantitative data exists on their skill sets in various areas. However, in the maintenance domain more data is prevalent, which can be used to assess skills of maintenance technicians. For example, Maintenance Work Orders (MWOs) contain a wealth of knowledge around various tasks and can provide insights into skill levels for different criteria. Open-source tools, such as Nestor^1^, can aid in annotating and analyzing MWOs (Sexton & Brundage, 2019). As an example, a technician may rate highly for plumbing skills, but low for electrical skills. As mentioned earlier, this assessment can be based on self assessment or by analyzing previous jobs completed by the maintainer.

### 4.4. Create Team

In this step, the team is formed using the information garnered from the above steps. Many algorithms exist to automate team formation. For example, CATME Team-Maker (Layton et al., 2010) uses a greedy randomized algorithm to form teams. The tool randomly creates teams of the desired size, and then iteratively reassigns members to maximize the minimum compliance score of the teams, where the compliance score represents the degree to which a team matches the desired composition specified when configuring the tool.

Hübscher describes another similar approach using the Tabu Search algorithm (Hübscher, 2010). Using this method, teams are generated by swapping potential team members until a local maximum (with respect to the desired criteria) is reached. However, the Tabu Search keeps track of previous moves and uses this knowledge to prevent the algorithm from retracing the same solution by repeatedly swapping the same members.

Other approaches focus on maximizing intra-group diversity and/or minimizing inter-group differences, such as (Baker & Powell, 2002; Beheshtian-Ardekani & Mahmood, 1986; Weitz & Lakshminarayanan, 1998). Additional examples include genetic algorithms (e.g., (Gogoulou, Gouli, Boas, Liakou, & Grigoriadou, 2007; Wang, Lin, & Sun, 2007)) and agent-based approaches (e.g., (Soh, Khandaker, & Jiang, 2006)). As an example, the team may consist of a maintenance manager, an experienced plumber, and a newly hired mechanic.

### 4.5. Solicit Feedback

The last step in the proposed workflow is to solicit feedback on the team formation process. This step was not as crucial in the original higher education context since student projects are often “one and done” and thus the feedback only informs the next batch of students. In the maintenance domain, however, the managers and non-managers are both likely to be reused in a new team formation. Soliciting feedback also allows employees to have another step to be involved in the team formation process, enabling more buy-in.

Managers often face a hard decision in choosing the best way to provide and communicate feedback in order to improve the quality of work. Prior research has investigated this issue; for example, one study analyzed the role of feedback by comparing the performance of three various feedback mechanisms during a contest for new product ideas (Wooten & Ulrich, 2017). The feedback mechanisms studied were: 1) no feedback, 2) random feedback, and 3) directed feedback, also known as in-process feedback. The study was performed where daily feedback was provided to the contestants and then consumers were asked to rate the quality of their product designs. The contestants were then grouped based on results of their performance and ideas. The result of the study showed that random feedback is better than no feedback. Moreover, it was evident that feedback as a whole does benefit the quality of ideas produced. However, directed feedback only had a positive impact if the quality of submission needed improvements; this was because it leads to less variance in product quality. (Wooten & Ulrich, 2017).

Regardless of how it is collected, the feedback gathered in this step of the proposed workflow could eventually feed directly into the team formation algorithm, allowing teams to be predicted for particular maintenance tasks. This step could be done, for example, by collecting preferences over time of how different people work together. Metrics like time needed to finish task, cost consumed, and several other points would feed into the algorithm as criteria or other constraints. As an example, the maintainer may want higher weights for engineering skill criteria versus the personality traits, which could impact future team creation.

### 4.6. Discussions

**Figure 2.**
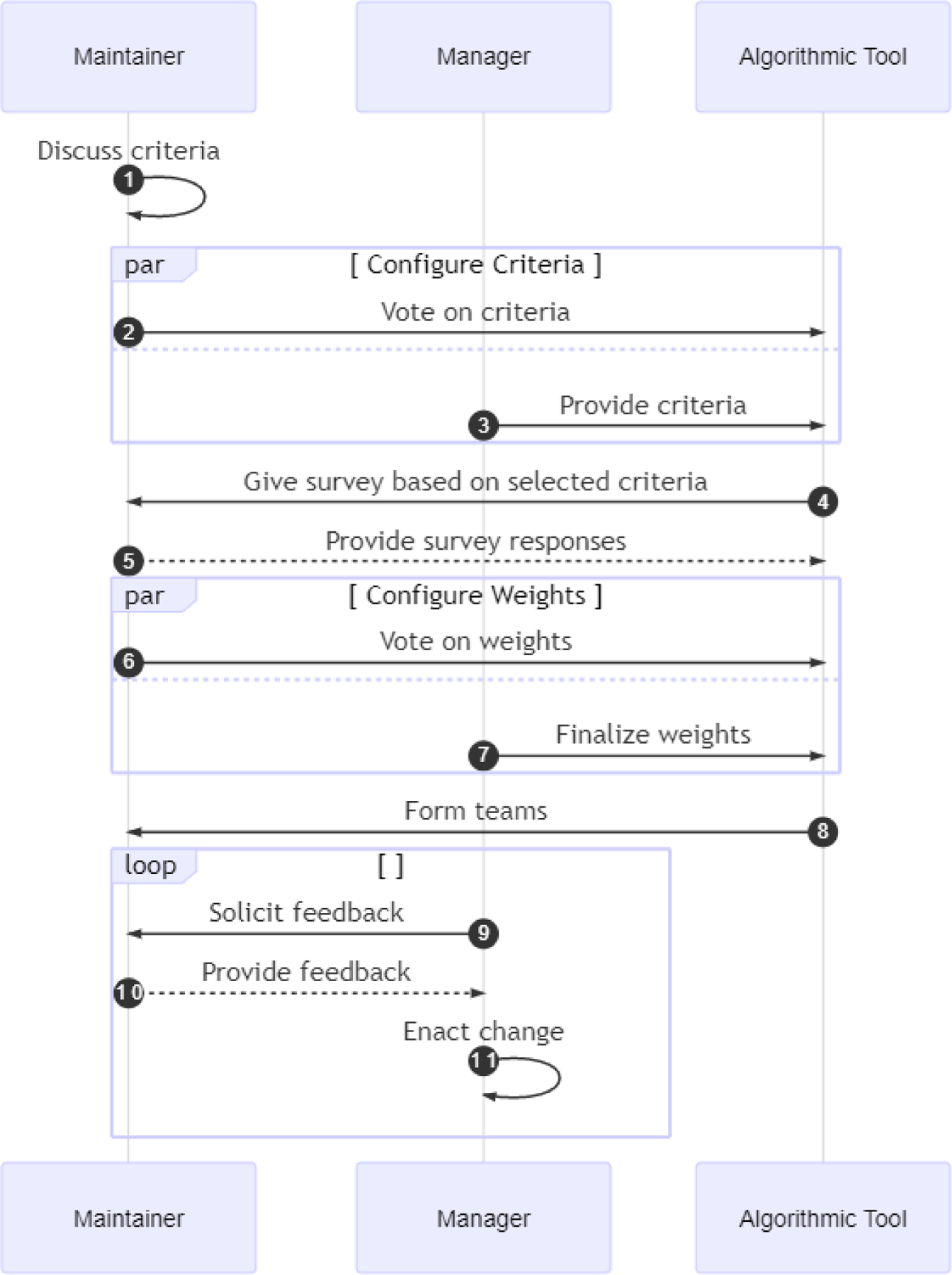
Sequence Diagram

This paper adapted the LIFT framework from its original context in academia to propose a team formation workflow more suited to the needs of maintainers. An overview of the steps discussed above is shown in Fig. 2.

While we describe 5 steps for effective team formation, the methods in which to implement these steps are flexible. For example, while the original LIFT used Likert scales for voting on criteria, this may not be the best solution for maintainers. The swim lane diagram in Fig. 2 shows the high-level steps, the different actors, and the information flows involved in the proposed workflow. Individual companies can substitute specific methods into each of these steps to best fit the needs of their particular maintenance contexts.

An effective team formation process, if performed correctly, will lead to more effective teams. As we discussed, this process allows involvement of all levels of the organization including management and technicians. This type of involvement leads to improved morale and organizational culture, which previous research indicates leads to improved productivity.

## 5. Conclusions and Future Work

This paper discusses the maintenance workflow and the team based activities in the process. A team formation procedure is presented derived and adapted from the LIFT framework for higher education. This process allows managers and non-managers to participate in the team formation process to improve the efficiency of these teams.

The workflow proposed in this paper is subject to some limitations. For example, as mentioned previously, this process may not be applicable or necessary in small companies, where there are only a few potential team members who may not vary widely relative to the selected criteria. In very large companies, it may be difficult to simultaneously satisfy the preferences of all maintainers and managers involved in the teams to be formed. Additional technological support may also be necessary to feasibly implement this process at a large scale, such as natural language processing techniques to assist with the extraction of criteria from the discussion phase, a task which was performed by hand in the original LIFT workflow (Hastings et al., 2020). The proposed methodology may also not be appropriate in situations where project requirements change frequently mid-process. Future work can investigate adaptations of this methodology that are better suited to projects with a high level of variability.

In future work, this process will be tested in an authentic maintenance environment to determine its effectiveness. The authors will work with Heating, Ventilation, and Air Conditioning (HVAC) maintenance teams to further develop the process. Another direction with this work is to integrate it into well established lean maintenance strategies to further improve adoption of this team formation strategy. Based on these future improvements, the proposed workflow could be developed into a standalone platform with a interface allowing easy adoption. We hope this paper encourages organizations to think of teamwork as a malleable resource; one that can be optimized to improve employee efficiency, learning, and morale.

## Figures and Tables

**Table 1.** A categorization of the potential criteria discussed for maintenance jobs, adapted from (Hastings et al., 2020).

Category	Example criteria
**Team**	
Team Management	Job function, Management role, Years in the company
Coordination Between Teams	Shift schedule, Different departments
Previous Teamwork Experiences	Previous projects
**Maintenance**	
Background	Years experience, Certifications, Education
Crystallized Knowledge	Trade skills, Maintenance skills
Commitment	Work-life balance requirements, Personal commitments
**Identity**	
Demographics	Race, Gender, Age
Personality/Interests	Personality type, Personal interests

**Table 2.** Sample Criteria and Weights.

Criterion	Weight
Schedule	2
Theoretical vs. hands-on	2
Plumbing skills	0
10+ years experience	−1
Programming skills	−2
Electrician skills	2
Management experience	1
Business acumen	0

## Footnotes

1
https://www.nist.gov/services-resources/software/nestor
